# Supplementary material for: Susceptibility Testing by Volatile Organic Compound Detection Direct from Positive Blood Cultures: A Proof-of-Principle Laboratory Study
Source: Antibiotics (Basel). 2022 May 24;11(6):705. doi: 10.3390/antibiotics11060705 (PMC9220186; doi:10.3390/antibiotics11060705)
Supplement: Supplementary file 1 [file antibiotics-11-00705-s001.zip › antibiotics-1698671-supplementary.pdf]

Supplementary Materials

Table S1. Samples excluded based on isolated micro-organisms other than *Enterobacterales* spp, *Staphylococcus* spp or *Enterococcus* spp.

Achromobacter xylosoxidans (3)
Acinetobacter baumannii (1)
Acinetobacter spp. (1)
Haemophilus influenzae (1)
Micrococcus luteus (2)
Neisseria meningitidis (1)
Pseudomonas aeruginosa (4)
Streptococcus agalactiae (1)
Streptococcus constellatus (milleri gr.) (1)
Streptococcus dysgalactiae (1)
Stenotrophomonas maltophilia (1)
Streptococcus milleri group (1)
Streptococcus vestibularis (salivarius gr.) (1)

Table S2. Specifications of errors occurred.

**Preparation errors SpecifAST®**	
Leaks	9 b/d in 4 blood cultures
Bacterial contamination	2 b/d in 1 blood culture
Incorrect number of antibiotic dilutions	2 b/d in 2 blood cultures
**Technical errors SpecifAST®**	
No signal	12 b/d in 4 blood cultures
Software problem	11 b/d in 4 blood cultures
Instrument error	3 b/d in 1 blood culture
**No bacterial growth in SpecifAST® and VITEK®2**	2 b/d in 1 blood culture

b/d: bug/drug combinations.

Table S3. Minimal inhibitory concentrations of *Enterobacterales, Staphylococcus* and *Enterococcus* spp. and corresponding tested antimicrobials measured by SpecifAST® and VITEK®2.

A. Ciprofloxacin, *Enterobacterales* spp.

		VITEK®2					
		**Susceptible**	**ATU**	**Resistant**			
		<=0.25	0.5	1	2	>2	**Total**
**SpecifAST®**	<=0.25	**35**	0	0	0	0	35
	0.5	0	0	0	0	0	0
	1	0	0	0	0	0	0
	2	0	0	0	0	0	0
	>2	0	0	0	0	**9**	9
	**Total**	35	0	0	0	9	**44**

B. Cefotaxime, *Enterobacterales* spp.

		VITEK®2									
		**Susceptible**			**I**	**Resistant**					
		<=0.25	0.5	1	2	4	8	16	32	>32	**Total**
**SpecifAST®**	<=0.25	**37**	0	0	0	0	0	0	0	0	37
	0.5	0	**1**	0	0	0	0	0	0	0	1
	1	0	0	0	0	0	0	0	0	0	0
	2	0	0	0	0	0	0	0	0	0	0
	4	0	0	0	0	0	0	0	0	0	0
	8	0	0	0	0	0	0	0	0	0	0
	16	0	0	0	0	**1**	0	0	0	0	1
	32	0	0	0	0	0	0	**1**	0	**1**	2
	>32	0	0	0	0	0	0	**1**	0	**3**	4
	**Total**	37	1	0	0	1	0	2	0	4	**45**

C. Meropenem, *Enterobacterales* spp.

		VITEK®2							
		**Susceptible**				**I**		**Resistant**	
		<=0.25	0.5	1	2	4	8	>8	**Total**
**SpecifAST®**	<=0.25	**43**	0	0	0	0	0	0	43
	0.5	**1**	0	0	0	0	0	0	1
	1	**1**	0	0	0	0	0	0	1
	2	0	0	0	0	0	0	0	0
	4	0	0	0	0	0	0	0	0
	8	0	0	0	0	0	0	0	0
	>8	0	0	0	0	0	0	0	0
	**Total**	45	0	0	0	0	0	0	**45**

D. Oxacillin, S. aureus, S. lugdunensis and S. saprophyticus.

		**VITEK®2**					
		**Susceptible**				**Resistant**	
		<=0.25	0.5	1	2	>2	**Total**
**SpecifAST®**	<=0.25	**6**	**3**	0	0	0	9
	0.5	0	**1**	0	0	0	1
	1	**1**	0	0	0	0	1
	2	0	0	0	0	0	0
	>2	**1**	0	0	0	0	1
	**Total**	8	4	0	0	0	**12**

E. Oxacillin, coagulase negative staphylococci other than *S. lugdunensis* and *S. saprophyticus.*

		**VITEK®2**					
		**Susceptible**	**Resistant**				
		<=0.25	0.5	1	2	>2	**Total**
**SpecifAST®**	<=0.25	**10**	0	0	0	**1**	11
	0.5	0	0	0	0	0	0
	1	**1**	0	**1**	0	0	2
	2	**1**	0	0	0	**4**	5
	>2	**1**	0	0	0	**8**	9
	**Total**	13	0	1	0	13	**27**

F. Cefoxitin (6 mg/L), *Staphylococcus* spp.

		**VITEK®2**		
		**Susceptible**	**Resistant**	
		Negative	Positive	**Total**
**SpecifAST®**	Negative	22	4	26
	Positive	0	12	12
	**Total**	22	16	**38**

G. Vancomycin, *S. aureus.*

		**VITEK®2**							
		**Susceptible**			**Resistant**				
		<=0.5	1	2	4	8	16	>16	**Total**
**SpecifAST®**	<=0.5	**4**	0	0	0	0	0	0	4
	1	**1**	**4**	0	0	0	0	0	5
	2	**2**	0	0	0	0	0	0	2
	4	**1**	0	0	0	0	0	0	1
	8	0	0	0	0	0	0	0	0
	16	0	0	0	0	0	0	0	0
	>16	0	0	0	0	0	0	0	0
	**Total**	8	4	0	0	0	0	0	**12**

H. Vancomycin, coagulase negative *Staphylococcus* spp.

		VITEK®2							
		**Susceptible**				**Resistant**			
		<=0.5	1	2	4	8	16	>16	**Total**
**SpecifAST®**	<=0.5	0	**1**	0	0	0	0	0	1
	1	**6**	**3**	**1**	0	0	0	0	10
	2	**3**	**3**	**1**	0	0	0	0	7
	4	**3**	**3**	**2**	0	0	0	0	8
	8	0	0	**1**	0	0	0	0	1
	16	0	0	0	0	0	0	0	0
	>16	0	0	0	0	0	0	0	0
	**Total**	12	10	5	0	0	0	0	**27**

I. Ampicillin, *Enterococcus* spp.

		VITEK®2					
		**Susceptible**		**I**	**Resistant**		
		<=2	4	8	16	>16	**Total**
**SpecifAST®**	<=2	**1**	0	0	0	0	1
	4	**2**	0	0	0	0	2
	8	**1**	0	0	0	0	1
	16	0	0	0	0	0	0
	>16	0	0	0	0	**5**	5
	**Total**	4	0	0	0	5	9

J. Vancomycin, *Enterococcus* spp.

		VITEK®2							
		**Susceptible**				**Resistant**			
		<=0.5	1	2	4	8	16	>16	**Total**
**SpecifAST®**	<=0.5	**5**	0	0	0	0	0	0	5
	1	**1**	**3**	0	0	0	0	0	4
	2	0	0	0	0	0	0	0	0
	4	0	0	0	0	0	0	0	0
	8	0	0	0	0	0	0	0	0
	16	0	0	0	0	0	0	0	0
	32	0	0	0	0	0	0	**1**	1
	>32	0	0	0	0	0	0	0	0
	**Total**	6	3	0	0	0	0	1	**10**

Interpretation of susceptibility test results according to EUCAST 2021 clinical breakpoints. Categorical agreement (green) Minor errors (orange) Major and very major errors (red). ATU: Area of technical uncertainty; I: Susceptible, increased exposure.

Table S4. Sensitivity and specificity of SpecifAST® method compared to VITEK®2.

**Gram negatives**	**Sensitivity (95%-CI)**	**Specificity (95%-CI)**	**Resistance prevalence**
Ciprofloxacin	100%	100%	9/45 (20%)
Cefotaxim	100%	100%	7/46 (19%)
Meropenem	100%	100%	0/46 (0%)
**Gram positives**			
Ampicillin	100%	75% (32.6 - 117.4)	8/12 (66.7%)
Oxacillin	92.9% (78.9 – 106.9)	85.7% (71.7 – 99.7)	14/42 (33.3%)
Cefoxitin	75% (53.8 - 96.2)	100%	16/41 (39.0%)
Vancomycin	100%	96.2% (91.0 - 101.4)	1/53 (1.9%)
**Mean**	91% (83.1 - 98.9)	97% (94.8 – 99.2)	55/285 (19.3%)

Sensitivity and specificity with corresponding 95% confidence intervals, and resistance prevalence. CI: Confidence interval.
